# Structural and functional analysis of cancer-associated missense variants in the retinoblastoma protein pocket domain

**DOI:** 10.1016/j.jbc.2025.108284

**Published:** 2025-02-10

**Authors:** Anthony Castro, Alfredo Ruiz Rivera, Chad C. Moorman, Emma R. Wolf-Saxon, Hailey N. Mims, Vanessa I. Vasquez Meza, Matthew A. Rangel, Marcos M. Loera, Ian C. Bond, Seth B. Buchanan, Estela Villarreal, Sarvind Tripathi, Seth M. Rubin, Jason R. Burke

**Affiliations:** 1 Department of Chemistry and Biochemistry, California State University, San Bernardino, California, USA; 2 Department of Chemistry and Biochemistry, University of California, Santa Cruz, California, USA

**Keywords:** cell cycle, crystal structure, E2F transcription factor, fluorescence anisotropy, protein stability, retinoblastoma protein (pRb, RB), tumor suppressor gene

## Abstract

The retinoblastoma tumor suppressor (Rb) is a multifunctional protein that primarily regulates the cell cycle but also has roles in cellular differentiation, DNA damage response, and apoptosis. The loss of Rb is a key event in the development or progression of many cancers. Essential functions of Rb occur through its pocket domain, which is necessary for regulating binding interactions with E2F transcription factors and transcription repressors that bind *via* an LxCxE motif. The pocket domain is the most highly conserved region of the multidomain protein, as well as the most frequent site of mutations. To understand what effects cancer missense mutations have on Rb’s pocket domain, we used fluorescence polarization and differential scanning fluorimetry to quantify changes caused by 75 cancer-associated missense variants to E2F transactivation domain (E2F^TD^) binding, LxCxE binding, and the thermostability of the pocket domain. We find that 43% of the missense variants tested reduce Rb-E2F^TD^ binding. Many of these variants are not located at the E2F^TD^-binding site, yet they destabilize the fold of the protein and show temperature-sensitive binding effects. We also find that 21% of tested mutations reduce LxCxE binding, and several mutations selectively disrupt either E2F^TD^ or LxCxE binding. Protein X-ray crystallography of four missense variants reveals how mutations destabilize the protein fold and inhibit E2F^TD^ or LxCxE binding. Taken together, this work provides the first understanding of the multiple ways through which stability, structure, and function of Rb’s pocket domain are altered by common missense mutations seen in cancer.

The retinoblastoma protein (Rb) is a multifunctional, chromatin-associated tumor suppressor protein. Functional Rb is critical for preventing the initiation of retinoblastomas, small cell lung cancers, and osteosarcoma, and Rb loss correlates with cancer progression of most other common human cancers (1). Recent reviews highlight the numerous context-dependent, noncanonical, and enigmatic roles of Rb and its deletion in cancers (2, 3, 4). However, Rb’s most critical role is as a key regulator of the cell cycle (5). One component of this regulation occurs late in G_1_ phase, when hyperphosphorylation of Rb by cyclin-dependent kinases disrupts repressive complexes between Rb and E2F, resulting in the activation of S-phase gene transcription (5). Phosphorylation causes structural changes to Rb that strongly inhibit E2F transactivation domain (E2F^TD^) binding (6, 7). Underscoring the importance of these inactivation mechanisms, Rb hyperphosphorylation is a common consequence of cancerous Rb pathway mutations (8). In certain contexts, cancer-associated missense mutations may be similar to the effects of persistent hyperphosphorylation of Rb, as they may enable retention of select functions of folded Rb while disrupting specific protein–protein interactions. This idea has been tested for some mutations which weaken Rb-E2F^TD^–binding interactions *in vitro* (9, 10) and *in vivo* (11). However, there is currently little known about the biochemical consequences of a majority of the observed cancer-associated missense mutations and to what extent these affect Rb–E2F and other Rb complexes.

Genome sequencing efforts have revealed detailed landscapes of somatic mutations in cancers (12). For a majority of these, clinical, cellular, biochemical, and structural outcomes remain unclear. Within the Rb protein, hundreds of cancer-associated missense mutations span multiple structured domains comprising conserved protein–protein interaction sites that regulate distinct functions (13, 14). Among these are mutations which potentially affect the structured LxCxE-binding cleft, which is responsible for recruiting corepressive complexes to silence E2F promoters (13). Mutation studies specifically targeting the LxCxE binding site have parsed some of Rb’s context-dependent tumor suppressive roles onto distinct regions of the protein. For example, mutations at the LxCxE-binding site do not affect cell cycle arrest, differentiation, or repression of E2F targets but instead prevent entry into senescence (15, 16). However, in the context of genotoxic stress caused by DNA damage, mutations specifically targeting the LxCxE-binding site do compromise repression of E2F targets and cell cycle control (15, 17, 18). Without genotoxic stress, a combination of mutations at multiple protein–protein interaction sites is needed to maximally impair Rb-mediated cell cycle control (16). The proteins that interact directly with Rb *via* LxCxE motifs include tumor suppressors ARID4A and KDM5A, as well as EID1 and others (19). Multiple oncogenic viruses have convergently evolved to target Rb *via* the LxCxE interface, suggesting an importance of the tumor suppressive functions at the site (20, 21). However, on the molecular and structural levels, it is still not entirely clear how Rb coordinates its multiple roles and which functions are lost that drive the initiation and evolution and specific cancers (2). Selected cancer mutations, M704V and R661W, have been shown to disrupt LxCxE-mediated protein binding, yet it is unclear whether other cancer mutations alter binding interactions as this site (10, 22).

Studies of specific cancer-associated Rb missense mutations have provided some insights into their consequences. The most well studied example, R661W, is a low-penetrance mutation in familial Rb that contributes partially to cell cycle arrest in retinoblastoma, is sufficient to initiate tumorigenesis, and causes cell cycle defects in embryos (23, 24, 25). Additionally, R661W, which alters both E2F and LxCxE binding, exhibits temperature sensitivity in a yeast growth assay, such that LxCxE binding is reduced at 30 °C and 37 °C when compared with 25 °C (10, 26). Temperature dependence is also a characteristic of some cancer-associated missense mutations in p53, where variants that destabilize p53 also disrupt binding to DNA, enhance degradation, and are drug targets for intervention by stabilizing compounds (27). Beyond R661W, however, for Rb, there has not been evidence of the consequences that cancer missense mutations have on thermostability and related changes to important protein–protein interactions (26).

The goal of this work is to use biochemical approaches to characterize the effects of a large set of the most common cancer-associated missense variants that occur to the pocket domain of the Rb tumor suppressor protein. By creating a large data set of binding and stability measurements of Rb missense variants, we seek to demystify predictive qualities about how these variants may differently affect Rb’s functions. The pocket domain was selected because it is the most frequent target of cancer mutations, it contains multiple functional binding interfaces, and it comprises a folded domain that is amenable to study through biochemical means. While a handful of cancer missense mutations to the pocket domain have been studied for E2F- and LxCxE-binding defects, there is currently little known about the biochemical consequences of the vast majority of Rb mutations associated with cancer (9, 10, 22). Here, we profile 75 Rb missense variants for changes to key binding interactions. The E7 oncoprotein from HPV has provided a structured example of binding at Rb’s LxCxE site and has been used extensively as a tool to study and characterize binding at this site (28, 29, 30). We use E7^LXCXE^ in order to understand effects that may occur to structurally similar, endogenous LxCxE-mediated binding interactions (19). In addition, we measure missense variant-induced changes to E2F1^TD^ binding, which serves as an indicator for binding changes to the homologous-activating E2Fs. Thermostability changes and temperature-sensitive binding effects are also measured, and together with protein X-ray crystallography, these experiments provide a comprehensive understanding of the biochemical consequences of cancer missense mutations to Rb’s pocket domain, as well as trends that define them.

## Results

### Expression of Rb pocket domain missense variants in *E. coli*

For this study, 112 missense variants in the Rb pocket domain (Rb^P^) were identified from two pan cancer genome somatic mutation databases, COSMIC (cancer.sanger.ac.uk) (31) and cBioPortal (cbioportal.org) (32). After cloning and expression attempts, we found that 37 missense variants could not be expressed from *E. coli*, indicating that protein misfolding or severe instability may be caused by these mutations (Fig. S1). The remaining 75 variants were successfully expressed and purified to homogeneity. The studied variants span all regions of the pocket domain, which includes subdomain A, subdomain B, and the unstructured 65-amino acid “pocket loop” (Rb^PL^) (Fig. S1).

### Disruptions to protein–protein interactions are a common consequence of cancer-associated Rb^P^ missense variants

**Figure 1:**
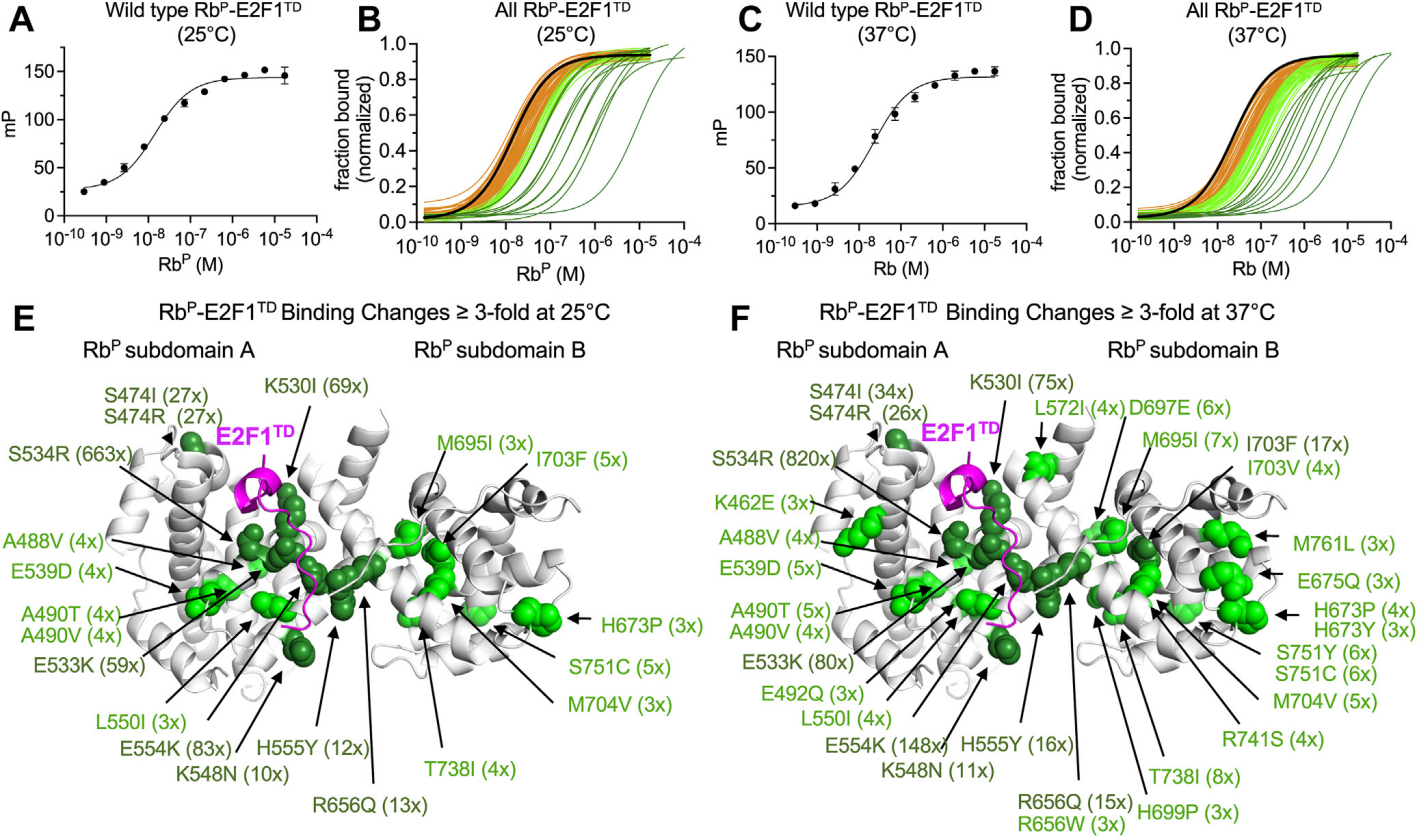
**Fluorescence polarization-binding experiments for Rb**^**P**^**-E2F1**^**TD**^**at 25 °C and 37 °C.***A*, the saturating binding curve for TMR-E2F1^TD^ binding to WT Rb^P^ at 25 °C. Error bars are SDs of four replicates. *B*, normalized binding curves for Rb^P^-E2F1^TD^ at 25 °C for WT Rb^P^ (*black*, n = 1); Rb^P^ variants which show less than three-fold increases to K_d (app)_ relative to WT (*orange*, n = 54); Rb^P^ variants which show 3- to 9-fold increases to K_d (app)_ relative to WT (*light green*, n = 12); and Rb^P^ variants which show 10-fold or greater increases to K_d (app)_ relative to WT (*dark green*, n = 9). *C*, the saturating binding curve for TMR-E2F1^TD^ binding to WT Rb^P^ at 37 °C. Error bars are SDs of four replicates. *D*, normalized binding curves for Rb^P^-E2F1^TD^ at 37 °C for WT Rb (*black*, n = 1); Rb^P^ variants which show less than three-fold increases to K_d (app)_ relative to WT (*orange*, n = 40); Rb^P^ variants which show 3- to 9-fold increases to K_d (app)_ (*light green*, n = 25); and Rb^P^ variants which show 10-fold or greater increases to K_d (app)_ relative to WT (*dark green*, n = 10). The structural positions of the missense variants with 3- to 9-fold increases to K_d (app)_ (*light green*, space-filling) and 10-fold or greater increases to K_d (app)_ (*dark green*, space-filling) are mapped onto the structure of Rb^P^-E2F1^TD^ (PDB: 1O9K) (37) for 25 °C binding experiments (*E*) and 37 °C binding experiments (*F*). Fold increases in K_d (app)_ relative to WT are shown in parentheses next to each mutation name.

Changes to binding affinities caused by missense variants were measured by fluorescence polarization (FP). In this FP assay, a peptide of the E2F1 transactivation domain that is N-labeled with tetramethyl rhodamine dye (TMR-E2F1^TD^) is measured in the presence of unlabeled Rb pocket (30). When the experiment is conducted at 25 °C, the measured equilibrium dissociation constant (K_d_) for E2F1^TD^ binding to WT Rb^P^ is 14 ± 3 nM (Fig. 1*A*, Table 1). This value is similar to the K_d_ value measured for WT Rb^P^-E2F1^TD^ by isothermal titration calorimetry (33). To examine Rb^P^ missense variants in this manner, we first considered that mutations to proteins tend to be destabilizing and can cause irreversible unfolding. In a binding assay, a tendency for unfolding can have the consequence of reducing the known concentration of active protein used for K_d_ measurements, necessitating the reporting of apparent K_d_ values (K_d (app)_), as opposed to K_d_ values (34, 35). In its WT form, the construct Rb^P^ is metastable when purified *in vitro*, and the protein is prone to temperature-dependent irreversible unfolding transitions (36). Taking this into consideration, we here report apparent equilibrium dissociation constant values (K_d (app)_) for each Rb^P^ variant. Through these measurements, we are able to compare changes to the binding capabilities of Rb^P^ missense variants. This type of comparison is particularly important for evaluating cancer-associated mutations at physiological temperature (37 °C), where effects of the variants are most relevant.

**Table 1:** K_D (app)_ and T_m_ data for all Rb^P^ variants and WT (∗denotes K_d_ value)

Rb^P^ variant		Rb^P^- E2F1^TD^ K_d (app)_ (nM)		Rb^P^- E7^LxCxE^ K_d (app)_ (nM)		Tm (°C)
		25 °C	37 °C	25 °C	37 °C	
WT		14 ± 3∗	22 ± 4	5 ± 1∗	12 ± 1	46.9 ± 0.5
1	E440K	12 ± 3	27 ± 4	5 ± 1	13 ± 1	44.2 ± 0.7
2	S443L	31 ± 6	55 ± 11	9 ± 1	21 ± 2	45.4 ± 0.3
3	Q444L	14 ± 3	30 ± 7	4 ± 1	11 ± 2	46.0 ± 0.8
4	Q444R	15 ± 3	25 ± 4	7 ± 2	17 ± 4	47.3 ± 0.6
5	R451C	33 ± 7	51 ± 9	7 ± 2	19 ± 4	46.4 ± 1.1
6	R451H	29 ± 6	56 ± 13	9 ± 2	22 ± 4	44.4 ± 0.7
7	R451S	26 ± 5	44 ± 9	9 ± 2	17 ± 2	45.5 ± 0.4
8	K462E	27 ± 4	67 ± 17	11 ± 2	32 ± 9	41.9 ± 0.2
9	K462T	30 ± 6	51 ± 10	8 ± 1	16 ± 3	47.2 ± 0.0
10	S463L	26 ± 5	41 ± 7	10 ± 7	17 ± 12	48.0 ± 0.8
11	S474I	364 ± 53	775 ± 148	7 ± 1	18 ± 2	43.7 ± 0.1
12	S474N	19 ± 5	26 ± 7	8 ± 3	13 ± 4	45.7 ± 0.3
13	S474R	365 ± 37	580 ± 63	5 ± 1	15 ± 3	44.4 ± 0.1
14	A488V	53 ± 11	78 ± 17	9 ± 1	19 ± 2	42.8 ± 0.3
15	A490S	17 ± 3	29 ± 5	7 ± 1	15 ± 1	46.1 ± 0.3
16	A490T	59 ± 13	115 ± 32	13 ± 2	33 ± 4	42.2 ± 0.9
17	A490V	48 ± 11	87 ± 20	11 ± 1	33 ± 3	41.8 ± 0.3
18	E492Q	22 ± 5	73 ± 18	14 ± 3	52 ± 12	41.5 ± 0.6
19	Q504E	18 ± 5	33 ± 9	5 ± 1	11 ± 2	46.9 ± 0.3
20	A525G	36 ± 7	65 ± 12	12 ± 4	52 ± 13	47.7 ± 0.4
21	K530I	945 ± 296	1680 ± 475	8 ± 3	20 ± 6	46.9 ± 0.2
22	E533K	810 ± 153	1805 ± 277	6 ± 1	14 ± 2	40.5 ± 0.1
23	S534R	9278 ± 2438	18,050 ± 3072	4 ± 2	11 ± 3	45.6 ± 0.3
24	E539D	55 ± 11	104 ± 17	7 ± 1	19 ± 2	42.0 ± 0.2
25	M546I	13 ± 3	21 ± 4	7 ± 1	13 ± 1	47.2 ± 0.2
26	K548N	133 ± 39	178 ± 56	6 ± 1	15 ± 3	46.5 ± 0.1
27	L550I	41 ± 6	80 ± 10	3 ± 1	7 ± 1	43.2 ± 0.5
28	E554K	1167 ± 98	3266 ± 722	12 ± 3	48 ± 9	39.9 ± 0.2
29	H555Y	158 ± 28	366 ± 72	8 ± 2	32 ± 6	44.4 ± 0.1
30	R556Q	22 ± 5	48 ± 12	5 ± 1	13 ± 2	46.7 ± 0.5
31	L572I	37 ± 12	87 ± 30	4 ± 2	15 ± 8	47.2 ± 0.7
32	D584Y	33 ± 7	51 ± 12	12 ± 2	20 ± 4	46.2 ± 0.1
33	H585Y	35 ± 8	92 ± 40	6 ± 1	18 ± 2	47.4 ± 0.8
34	S588F	36 ± 8	51 ± 12	5 ± 1	16 ± 1	46.0 ± 0.1
35	P595L	46 ± 9	83 ± 12	14 ± 3	32 ± 6	46.8 ± 0.2
36	D604G	27 ± 5	50 ± 11	13 ± 5	26 ± 9	46.2 ± 0.7
37	Y606C	15 ± 3	27 ± 4	7 ± 4	23 ± 11	45.4 ± 0.3
38	R611S	15 ± 4	30 ± 11	5 ± 1	15 ± 5	46.7 ± 0.1
39	S612P	32 ± 6	49 ± 11	7 ± 2	17 ± 3	45.5 ± 0.1
40	T620M	24 ± 8	59 ± 16	6 ± 2	15 ± 5	46.1 ± 1.1
41	R621C	22 ± 4	43 ± 7	12 ± 3	30 ± 5	45.8 ± 0.0
42	R621H	30 ± 5	48 ± 8	10 ± 3	18 ± 4	46.9 ± 0.1
43	R621P	40 ± 8	83 ± 20	12 ± 2	32 ± 6	45.9 ± 0.6
44	R621S	21 ± 5	38 ± 9	6 ± 1	17 ± 3	45.9 ± 0.4
45	T638S	14 ± 2	38 ± 9	7 ± 1	14 ± 3	46.5 ± 0.2
46	S648T	33 ± 9	58 ± 14	6 ± 1	14 ± 3	46.6 ± 0.2
47	V654L	18 ± 3	30 ± 4	11 ± 2	21 ± 2	48.2 ± 0.1
48	R656Q	183 ± 36	325 ± 75	6 ± 1	14 ± 1	46.5 ± 0.1
49	R656W	39 ± 7	77 ± 11	8 ± 2	19 ± 3	43.1 ± 0.2
50	T664A	15 ± 2	24 ± 4	8 ± 4	16 ± 5	46.6 ± 0.1
51	R668C	12 ± 4	25 ± 10	3 ± 1	16 ± 3	44.9 ± 0.6
52	R668H	13 ± 2	22 ± 4	7 ± 3	28 ± 7	44.2 ± 0.1
53	S671F	24 ± 5	46 ± 11	9 ± 1	19 ± 2	46.1 ± 0.2
54	H673P	42 ± 6	98 ± 15	24 ± 2	83 ± 6	43.3 ± 0.8
55	H673Y	26 ± 5	66 ± 13	10 ± 1	29 ± 3	45.2 ± 0.0
56	E675Q	35 ± 8	71 ± 21	20 ± 9	46 ± 18	46.4 ± 0.1
57	I679F	17 ± 4	33 ± 10	11 ± 5	20 ± 10	45.9 ± 0.1
58	H686Y	20 ± 4	35 ± 7	5 ± 1	14 ± 2	46.6 ± 0.5
59	T687I	20 ± 4	40 ± 6	13 ± 1	29 ± 2	48.9 ± 0.3
60	E693A	42 ± 6	59 ± 18	7 ± 1	24 ± 3	43.1 ± 0.2
61	M695I	43 ± 10	165 ± 33	11 ± 6	93 ± 44	43.8 ± 0.2
62	D697E	29 ± 6	129 ± 35	9 ± 2	46 ± 8	42.8 ± 0.2
63	H699P	35 ± 6	68 ± 14	6 ± 1	14 ± 2	43.6 ± 0.2
64	I703F	63 ± 12	396 ± 12	16 ± 2	275 ± 26	40.3 ± 0.4
65	I703V	20 ± 4	99 ± 22	21 ± 11	53 ± 26	43.7 ± 0.2
66	M704V	41 ± 8	129 ± 37	18 ± 4	51 ± 16	41.8 ± 0.7
67	T738I	60 ± 11	179 ± 34	18 ± 2	151 ± 24	41.6 ± 0.1
68	R741C	23 ± 3	64 ± 12	14 ± 3	23 ± 2	44.3 ± 0.2
69	R741H	21 ± 4	41 ± 7	18 ± 6	21 ± 4	45.8 ± 0.1
70	R741S	32 ± 7	84 ± 23	16 ± 1	46 ± 5	43.7 ± 0.4
71	S751C	66 ± 13	137 ± 32	44 ± 7	98 ± 19	46.0 ± 0.4
72	S751Y	33 ± 5	127 ± 25	127 ± 8	420 ± 38	41.5 ± 0.2
73	V754G	20 ± 4	34 ± 7	7 ± 2	15 ± 2	44.6 ± 0.3
74	M761L	32 ± 7	68 ± 13	1.3 ± 0.4	5 ± 1	46.4 ± 0.3
75	R763T	37 ± 11	59 ± 18	21 ± 6	35 ± 7	45.3 ± 0.3

Reported errors in the Kd or apparent Kd from FP measurements are curve-fitting errors.

When the Rb^P^-E2F1^TD^–binding experiment is conducted using 75 missense variants of Rb^P^ at 25 °C, we observe that 28% of the variants tested (n = 21) have three-fold, or more, weaker binding to E2F1^TD^ relative to WT (Fig. 1*B*, Table 1, Figs. S2–S6). Although *in vitro* binding assays are most often conducted under standard biochemical temperature at 25 °C, the effects of certain cancer-associated mutations may be observable only at physiological temperature (37 °C). To address this possibility, the experiments were conducted again at 37 °C. For WT Rb^P^ binding to E2F1^TD^, the measured apparent equilibrium dissociation constant (K_d (app)_) at physiological temperature is 22 ± 4 nM (Fig. 1*C*, Table 1). When the same experiment is conducted using 75 Rb^P^ missense variants, we observe that 46% of the variants tested (n = 35) cause three-fold, or more, weaker E2F1^TD^ binding relative to WT (Fig. 1*D*, Table 1, Figs. S2–S6). These data sets overlap, such that 20 of the 21 variants with three-fold or greater effects at 25 °C also have three-fold or greater effects at 37 °C (Fig. S7). The large percentage of tested mutations that are found to weaken Rb^P^ binding to E2F1^TD^ reveals how broadly susceptible this important binding interaction is to disruptions caused by cancer-associated missense mutations in Rb^P^.

Trends emerge when the positions of missense variants are mapped onto the structure of Rb’s pocket domain. At 25 °C, the majority of variants that strongly reduce E2F1^TD^ binding (by 10-fold or greater) are located at the E2F^TD^-binding cleft; these include S534R (820-fold), K530I (63-fold); E533K (59-fold); E544K (34-fold); R656Q (13-fold); H555Y (12-fold); and K548N (10-fold) (Fig. 1*E*). In the X-ray structures of Rb^P^-E2F1^TD^ and Rb^P^-E2F2^TD^, each of the WT forms of these seven amino acids forms binding interactions with E2F^TD^ (37, 38). Two variants show greater than 10-fold reductions in binding to E2F1^TD^ relative to WT yet do not make structured contacts with E2F^TD^: S474R (27-fold); and S474I (27-fold). At 37 °C, several additional variants in structured areas outside of the E2F^TD^-binding cleft also weaken Rb-E2F^TD^ binding (Fig. 1*F*). These occur within the hydrophobic core of subdomain A (A488V, A490S, A490T, L550I) and the hydrophobic core of subdomain B (M695I, I703F, I703V, M704V), as well as within other regions of the protein fold. Within the hydrophobic core of subdomain B, the variant I703F has a strong temperature-dependent effect on weakening E2F1^TD^ binding. For I703F, E2F1^TD^ binding is weakened by approximately five-fold relative to WT at 25 °C, yet this effect increases to 17-fold at 37 °C (Fig. 1, *E* and *F*, Table 1). Together, these results reveal the large extent of E2F^TD^ binding loss caused by cancer-associated missense variants. This analysis further reveals that E2F^TD^ binding loss is caused by variants located throughout the structured region of Rb^P^ and not limited to those located at the E2F^TD^-binding cleft.

**Figure 2:**
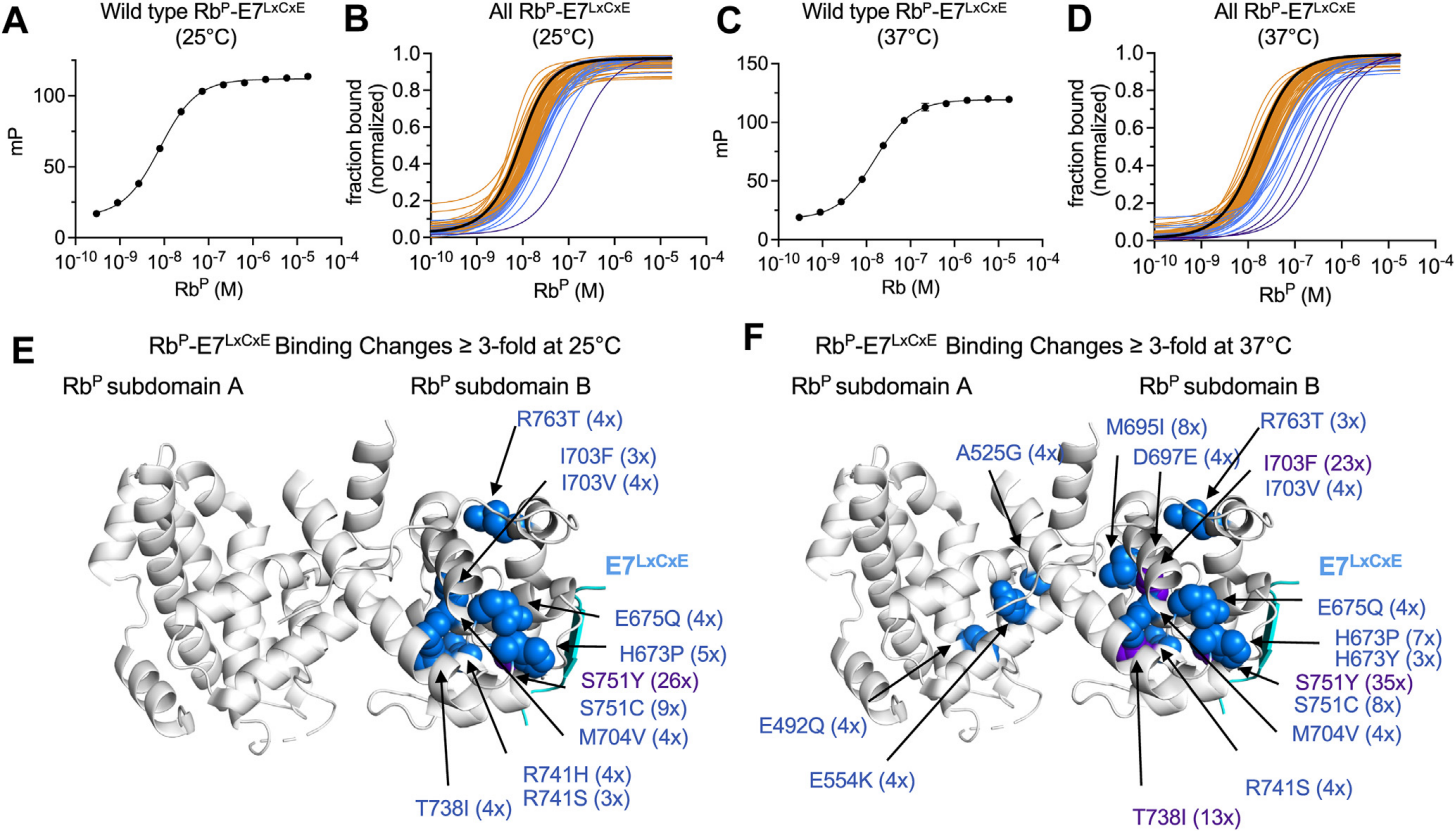
**Fluorescence polarization-binding experiments for Rb**^**P**^**-E7**^**LxCxE**^**at 25 °C and 37 °C.***A*, the saturating binding curve used to measure binding between TMR-E7^LxCxE^ and WT Rb^P^ at 25 °C. Error bars are SDs of four replicates. *B*, normalized binding curves measured at 25 °C depict binding between E7^LxCxE^ and WT Rb^P^ (*black*, n = 1); Rb^P^ variants with less than three-fold increases to K_d (app)_ relative to WT (*orange*, n = 63); Rb^P^ variants with 3- to 9-fold increases to K_d (app)_ relative to WT (*blue*, n = 11); and Rb^P^ variants which show 10-fold or greater increases to K_d (app)_ relative to WT (*purple*, n = 1). *C*, the saturating binding curve for TMR-E7^LxCxE^ binding to WT Rb^P^ at 37 °C. Error bars are SDs of four replicates. *D*, for binding at 37 °C, normalized binding curves are shown for E7^LxCxE^ binding to WT Rb (*black*, n = 1); Rb^P^ variants with less than three-fold increases in K_d (app)_ relative to WT (*orange*, n = 59); Rb^P^ variants with 3- to 9-fold increases in K_d (app)_ relative to WT (*blue*, n = 13); and Rb^P^ variants which show 10-fold or greater increases to K_d (app)_ relative to WT (*purple*, n = 3). The structural positions of the missense variants with 3- to 9-fold increases to K_d (app)_ (*blue*, space-filling) and 10-fold or greater increases to K_d (app)_ (*purple*, space-filling) are mapped onto the structure of Rb^P^-E7^LxCxE^ (PDB: 1GUX) (38) for binding experiments conducted at 25 °C (*E*) and 37 °C (*F*). Fold increases in K_d (app)_ relative to WT are shown in parentheses next to each mutation name.

To evaluate the Rb^P^-E7^LxCxE^ protein–protein interaction, the equilibrium dissociation constant for WT Rb^P^ binding to TMR-E7^LxCxE^ was measured to be K_d_ = 5 ± 1 nM (Fig. 2*A*, Table 1). This value is similar to the reported K_d_ between Rb^P^ and FITC-E7^LxCxE^ (29). When this experiment is conducted with 75 missense variants of Rb^P^, 15% of the variants tested (n = 11) have a K_d (app)_ approximately three-fold higher, or more, than the K_d_ for WT (Fig. 2 *B*, Table 1, Figs. S2–S6). At 37 °C, WT Rb^P^ binds to E7^LxCxE^ with K_d (app)_ = 12 ± 1 nM (Fig.2*C*, Table 1). At physiological temperature, 21% of the variants tested (n = 16) have a three-fold higher, or more, K_d (app)_ than wild type (Fig. 2*D*, Table 1, Figs. S2–S6). When the mutation sites are mapped onto the structure of Rb^P^-E7^LxCxE^, all of the mutation sites negatively affecting E7^LxCxE^ binding at 25 °C sit within subdomain B, close to the LxCxE-binding site (Fig. 2*E*). At 37 °C, additional sites within subdomain B have greater than three-fold effects, as do three sites outside of subdomain B: E492Q; E554K; and A525G (Fig. 2*F*). The biggest reduction in E7^LxCxE^ binding occurs for S751Y, which weakens the Rb^P^–E7^LxCxE^ binding interaction relative to WT by approximately 26-fold at 25 °C and 35-fold at 37 °C (Fig. 2, *E* and *F*). The strongest temperature-dependent effects reduce Rb^P^-E7^LxCxE^ binding relative to WT by up to several fold more at 37 °C compared to 25 °C. This is strongest for I703F, which reduces Rb^P^-E7^LxCxE^ binding approximately three-fold at 25 °C and approximately 23-fold at 37 °C (Fig. 2, *E* and *F*, Table 1). Because the molecular determinants of Rb^P^-LxCxE–binding interactions are similar across proteins, the mutations identified here as those that weaken Rb^P^-E7^LxCxE^ are likely to also weaken other LxCxE interactions mediating repressive complexes (19, 39).

### Protein instability in Rb^P^ is caused by cancer-associated missense variants

**Figure 3:**
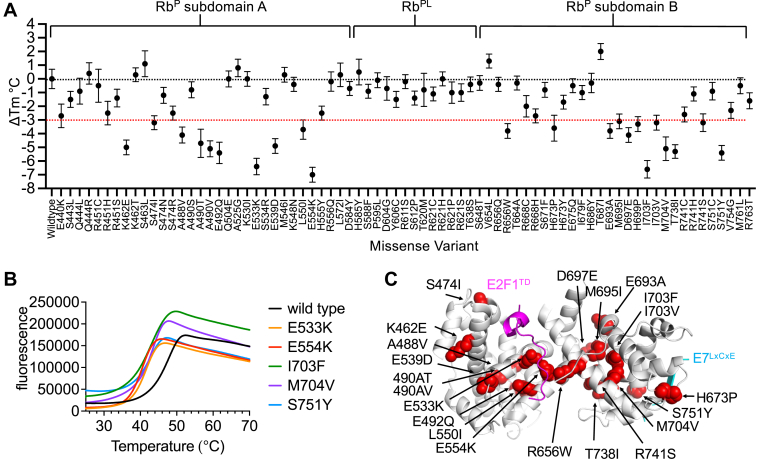
**Changes to the thermostability of Rb**^**P**^**caused by missense variants.***A*, differences between the T_m_ values of Rb^P^ variants relative to WT Rb^P^ (ΔT_m_) reveal the relative size of destabilizing effects caused by each variant. Error bars are propagated from SDs of averages calculated from nine replicates for WT and each missense variant. *B*, examples of raw fluorescence data for DSF runs of WT Rb^P^ and four example variants which show some of the greatest destabilizing effects. *C*, missense variants that cause a negative shift in T_m_ greater than a 3 °C threshold mapped onto the structure of Rb^P^.

WT Rb and missense variants were evaluated for stability changes by measuring the melting temperature (T_m_), defined as the temperature at which 50% of a protein sample is unfolded (40). When WT Rb^P^ undergoes irreversible melting *via* differential scanning fluorimetry (DSF), a T_m_ value of 46.9 ± 0.5 °C is calculated from a Boltzmann fit of the melting curve (Table 1). This value is similar to those from experiments assessing the stability of Rb pocket domain constructs by circular dichroism: T_m (app)_ = 49 °C and T_m_ = 46 °C (36, 41). Examination of Rb^P^ missense variants by DSF reveals a remarkable trend: nearly all tested variants in the A and B subdomains destabilize the pocket domain, and approximately 30% (n = 30) of all structured variants tested have larger than −2 °C reductions in T_m_, as measured by the difference between variant T_m_ and WT T_m_ values. None of the Rb^PL^ variants show greater than −2 °C reductions in T_m_ (Fig. 3*A*, Table 1). This is reasonable because the unstructured pocket loop does not contribute meaningfully to the stability of the folded pocket domain. Within the cyclin fold of subdomain A, the greatest destabilizing effects (ΔT_m_) are caused by E554K (−7.0 ± 0.5 °C), E533K (−6.4 ± 0.5 °C), E492Q (−5.4 ± 0.8 °C), A490V (−5.1 ± 0.6 °C), K462E (−5.0 ± 0.5 °C), E539D (−4.9 ± 0.5 °C), A490T (−4.7 ± 1.0 °C), and A488V (−4.1 ± 0.6 °C) (Fig. 3*A*, Table 1). Within subdomain B, the greatest destabilizing effects are caused by I703F (−6.6°C ± 0.6 °C), S751Y (−5.4 ± 0.5 °C), T738I (−5.3 ± 0.5 °C), M704V (−5.1 ± 0.9 °C), and D697E (−4.1 ± 0.5 °C) (Fig. 3*A*, Table 1). Examples of fluorescence traces used to calculate and evaluate the T_m_ values for WT Rb^P^, E533K, E554K, I703F, M704V, and S751Y are shown (Fig. 3*B*). Missense variants that pass a threshold of ΔT_m_ ≤ −3 °C relative to WT were mapped on to the structure of Rb^P^ (Fig. 3*C*). Several of the greatest destabilizing variants occur at amino acid sites completely buried within the hydrophobic cores of subdomain A (A488, A490, I550) and subdomain B (M695, I703, M704). It is also notable that all of the mutations which cause destabilizing effects of ΔT_m_ ≤ −3 °C also weaken Rb^P^ binding to E2F1^TD^ by three-fold or more (Figs. 1*F* and 3*C*).

### The relationship between T_m_ and temperature-sensitive binding for Rb^P^ variants

**Figure 4:**
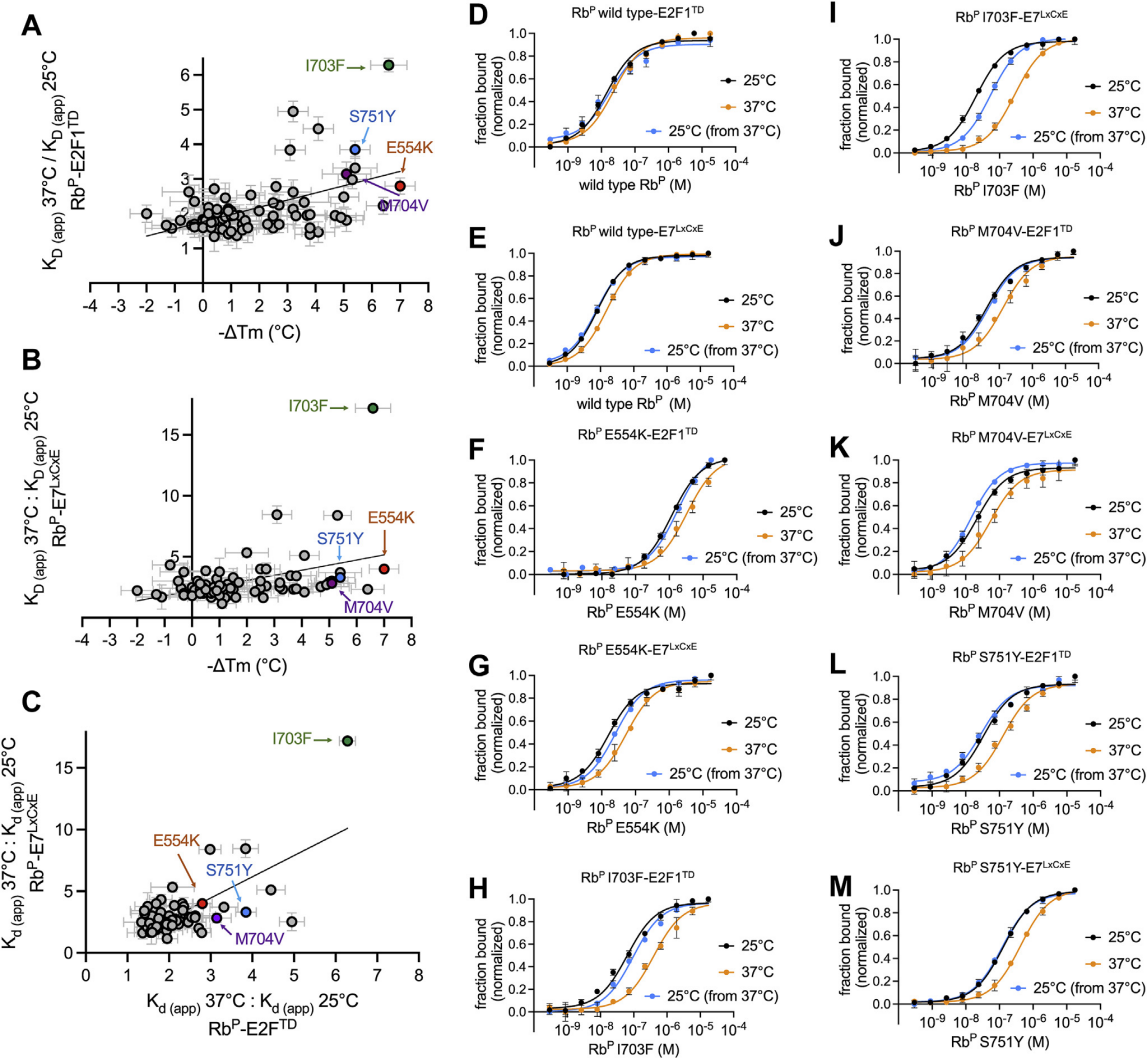
**Temperature-sensitive binding effects of Rb**^**P**^**missense variants.** Scatter plot of the ratio of K_d (app)_ values (37 °C: 25 °C) for E2F1^TD^ (*A*) and E7^LxCxE^ (*B*), as a function of -ΔT_m_. Scatter plot of the ratio of K_d (app)_ values (37 °C: 25 °C) for E7^LxCxE^ as a function of the ratio of K_d (app)_ values (37 °C: 25 °C) for E2F1^TD^ (*C*). Error bars are propagated from FP SDs and DSF SDs between four and nine replicates, respectively. The data were fit to a linear regression: y = 0.04081x+0.1125 with r^2^ = 0.26 for (*A*), y = 0.1073x+0.2958 with r^2^ = 0.17 for (*B*), and y = 1.717x-0.6978 with r^2^ = 0.48 for (*C*). Fluorescence polarization experiments were performed to assess the reversibility of temperature-dependent binding changes to E2F1^TD^ and E7^LxCxE^, respectively, for Rb^P^ WT (*D*, *E*), E554K (*F*, *G*), I703F (*H*, *I*), M704V (*J*, *K*), S751Y (*L*, *M*). The experiments measured binding initially at 25 °C (*black*), then at 37 °C (*orange*), and also after cooling down to 25 °C from 37 °C (*blue*). Error bars are SDs of four replicates.

To explore the possibility that missense variants which cause greater destabilizing effects also disrupt binding to E2F1^TD^ to a greater extent at higher temperatures, a plot was made of ΔT_m_ values (x-axis) *versus* the ratio of measured K_d (app)_ values for Rb^P^-E2F1^TD^ at 37 °C and 25 °C (y-axis) (Fig. 4*A*). A linear fit of this scatter plot reveals a positive association between the -ΔT_m_ change and the quotient of K_d (app)_ 37 °C/K_d (app)_ 25 °C. A similar relationship is also seen for Rb^P^-E7^LxCxE^ (Fig. 4*B*). A similar plot comparing K_d (app)_ 37 °C/K_d (app)_ 25 °C for Rb^P^-E2F1^TD^ versus Rb^P^-E7^LxCxE^ reveals a positive correlation for temperature-dependent changes to binding, indicating that many mutations impact both binding sites through alterations in protein stability (Fig. 4*C*). From this analysis, the variant I703F stands out as being the most temperature sensitive for both E2F1^TD^ and E7^LxCxE^ binding; however, variants from other regions of the protein, E554K, M704V, and S751Y, also follow the trend. In general, these relationships may be useful to predict variants that are temperature-sensitive based only on their T_m_ values, or alternatively, multitemperature-binding data may be used to identify variants that are likely destabilizing. This approach may be particularly useful for identifying cryptic disease-associated variants that are less thermostable yet do not significantly weaken binding interactions at 25 °C and may be more likely to have effects at 37 °C.

One explanation for the observed relationship between the measured ΔT_m_ values and the temperature-sensitive binding effects is that less stable variants become irreversibly unfolded at higher temperatures, creating conditions that appear as weaker binding. To explore this, four variants were selected from different regions of Rb^P^ to examine the reversibility of temperature-dependent effects of E2F1^TD^ and E7^LxCxE^ binding. For each variant, the K_d (app)_ is measured initially at 25 °C. After temperature ramping and incubating at 37 °C, the K_d (app)_ is measured again. To explore reversibility, a third measurement of K_d (app)_ is taken of a sample that is ramped to 37 °C, incubated at 37 °C, then cooled down again to 25 °C. The saturating binding curves produced under these three conditions reveal that in each case, the prewarming and postwarming measurements at 25 °C are each very close to one another, while the measurement at 37 °C is significantly different (Fig. 4, *D*–*M*). The K_d (app)_ values for Rb^P^ binding to E2F1^TD^ at 25 °C, measured after warming to 37 °C then cooling to 25 °C, are 17 ± 5 nM for WT; 1737 ± 224 nM for E554K; 94 ± 13 nM for I703F; 46 ± 9 nM for M704V; and 28 ± 8 nM for S751Y. The K_d (app)_ values for Rb^P^ binding to E7^LxCxE^, measured warming to 37 °C then cooling to 25 °C, are 7 ± 1 nM for WT; 24 ± 2 nM for E554K; 53 ± 6 nM for I703F; 14 ± 1 nM for M704V; and 119 ± 12 nM for S751Y. Reported errors in the K_d (app)_ measurements are curve-fitting errors. The other values for K_d (app)_, measured at 25 °C and 37 °C, shown in Figure 4, *D*–*M* are given in Table 1; comparative K_d (app)_ values calculated from the plots shown in Fig. 4, *D*–*M* are shown in Tables S1 and S2. Together, these results show that weaker binding at 37 °C is completely reversible for WT, E554K, M704V, and S751Y, as tested under these conditions. The exception is I703F, which upon cooling back down to 25 °C from 37 °C, binds weaker to E7^LxCxE^ (K_d (app)_ 53 ± 6 nM) than it does when initially measured at 25 °C (K_d (app)_ 16 ± 2 nM). These data suggest that I703F unfolds irreversibly to some extent at physiological temperature, while other missense variants exhibit reversible fold destabilizations which may underly the temperature sensitivity of binding. Future studies of binding enthalpies will be needed to provide more complete descriptions of thermodynamic relationships underlying the effects of Rb^P^ variants.

### X-ray crystal structures reveal bases of cancer mutation effects

**Figure 5:**
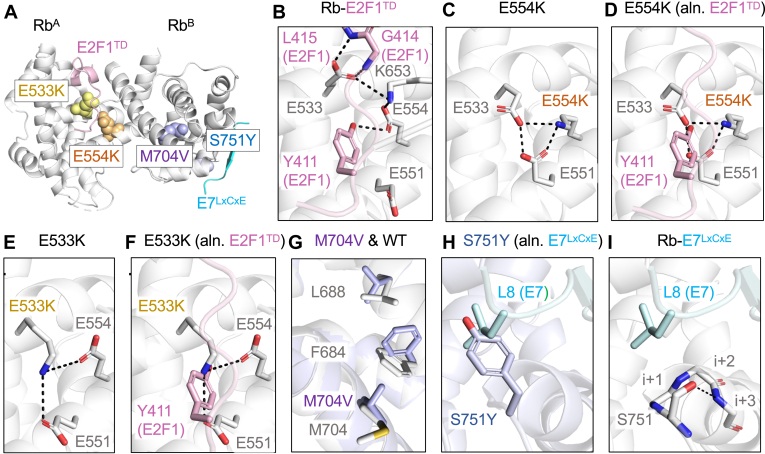
**Protein X-ray crystal structures of missense variants and comparisons to WT Rb**^**P**^**structures.***A*, the locations of crystallized variants in the pocket domain relative to E2F1^TD^- and E7^LxCxE^-binding sites. *B*, WT Rb^P^-E2F1^TD^ binding interactions are mediated by E533 and E554, from a previously published structure (PDB: 1O9K) (37). *C*, the structure of E554K shows the formation of a salt bridge network involving E554K, E533, and E551. *D*, a structural alignment of the WT Rb^P^-E2F1^TD^ with the structure of E554K reveals that the E554K-mediated salt bridge network blocks an important tyrosine (Y411) binding site of E2F^TD^. *E*, the structure of E533K shows the formation of a salt bridge network involving E533K, E554, and E551, similar to the E554K structure. *F*, a structural alignment of the WT Rb^P^-E2F1^TD^ with the structure of E533K reveals that the salt bridge network created by the missense variant blocks the tyrosine (Y411) binding site of E2F^TD^, similar to the effect of E554K. *G*, the structure of M704V reveals how the mutation induces a change in the packing of the hydrophobic core of subdomain B. *H*, the structure of S751Y aligned to the structure of Rb^P^-E7^LxCxE^ shows how the tyrosine variant occupies the binding pocket for L8 in E7^LxCxE^ peptide (PDB: 1GUX) (28). *I*, the previously published structure of WT Rb^P^-E7^LxCxE^ reveals S751 forms an H-bond to stabilize an ⍺-helix, the loss of which may contribute to the reduced stability of S751Y (PDB: 1GUX) (28).

For characterization by protein X-ray crystallography, four variants were studied: E533K, E554K, M704V, and S751Y. In order to crystalize these variants, each missense mutation was introduced into a modified Rb^P^ construct previously used to crystalize the Rb^PL^–Rb^P^ interaction (42). X-ray diffraction data collected from the crystals were used to solve the structures of the four variants by molecular replacement (Table 2). These specific variants were selected because they cause some of the greatest decreases in thermostability (ΔT_m_ ≤ −5 °C), strongly disrupt E2F1^TD^ or E7^LxCxE^ binding with temperature-dependent effects, and map to key regions of the pocket domain (Fig. 5*A*).

**Table 2:** X-ray crystallography data collection and refinement statistics

Crystal name	Rb E533K	Rb E554K	Rb M704V	Rb S751Y
Data collection				
Space group	R 3:H	R 3:H	R 3:H	R 3:H
Cell dimensions				
*a*, *b*, *c* (Å)	250.14, 250.14, 35.31	250.36, 250.36, 35.31	250.84, 250.84, 35.39	255.04, 255.04, 35.36
*α*, *β*, γ (°)	90, 90, 120	90, 90, 120	90, 90, 120	90, 90, 120
Resolution (Å)	47.27–2.16	47.31–2.26	47.4–2.38	48.2–2.32
*R*_merge_	0.191 (2.665)	0.215 (2.575)	0.233 (3.180)	0.174 (2.774)
*R*_pim_	0.092 (1.284)	0.104 (1.301)	0.079 (1.130)	0.085 (1.382)
CC ½	0.997 (0.401)	0.997 (0.369)	0.997 (0.381)	0.998 (0.302)
*I*/σ*I*	9.9 (1.1)	9.6 (1.0)	10.2 (0.9)	12.4 (0.9)
Completeness (%)	100.0	100.0	100.0	100.0
Multiplicity	10.4	10.4	10.5	10.5
Refinement				
Resolution (Å)	41.69–2.16 (2.237–2.16)	41.73–2.26 (2.314–2.26)	34.78–2.38 (2.465–2.38)	48.2–2.32 (2.403–2.32)
No. of unique reflections	44,174 (4393)	38,642 (3919)	33,256 (3275)	37,130 (3729)
*R*_work_/*R*_free_	0.2260 (0.3148)/0.2623 (0.3373)	0.2273 (0.3286)/0.2653 (0.3725)	0.2203 (0.3313)/0.2772 (0.3737)	0.2126 (0.3641)/0.2629 (0.4060)
No. of atoms	5753	5791	5694	5793
Protein	5679	5653	5685	5715
Water	74	138	9	78
B-factors (Å^2^)				
Average	66.51	58.99	71.70	76.89
Protein	66.68	59.24	71.71	77.24
Water	53.25	48.61	62.82	50.88
R.m.s.d.				
Bond lengths (Å)	0.015	0.016	0.015	0.015
Bond angles (°)	1.50	1.41	1.51	1.50
Ramachandran Plot				
Favored (%)	98.51	96.71	96.73	97.33
Allowed (%)	1.49	3.14	3.12	2.67
Outliers (%)	0.00	0.15	0.15	0.00
PDB codes	9DHU	9DHF	9DGK	9DHC

Parentheses are for the highest resolution shell.

For E554K and E533K, we sought to understand the structural basis for large reductions in Rb^P^-E2F1^TD^ binding as well as substantial decreases in thermostability. The significance of E554 in Rb-E2F^TD^ binding was initially pointed out by the authors of the two Rb^P^-E2F^TD^ structures as forming an H-bond the phenolic hydroxyl of conserved tyrosine in E2F1^TD^ and E2F2^TD^ (Fig. 5*B*) (37, 38). Additionally, from the structure of Rb^P^-E2F1^TD^, the sidechain of E553 forms two H-bonds with the backbone amides of L415 and G414 of E2F1^TD^ (Fig. 5*B*). These structured interactions show the roles of glutamate sidechains, E553 and E554, in mediating binding interactions between WT Rb^P^ and E2F^TD^. The structure of the cancer-associated variant, E554K, reveals how the lysine substitution disrupts the potential for these interactions by forming a shared salt bridge with E533 and E551 (Fig. 5*C*). This high-energy intramolecular bond does two notable things that likely disrupt E2F^TD^ binding: 1) it stabilizes a rotamer of E533 in an orientation is not amenable to E2F^TD^ backbone binding and 2) it places E533 and E551 in a triad with E554K that sterically occludes the binding pocket occupied by Y411 (Fig. 5*D*). Remarkably, the crystal structure of E533K shows something similar in which the mutated lysine sidechain creates a salt bridge triad with E554 and E551 in a manner that occludes the Y411(E2F1^TD^)-binding pocket (Fig. 5, *E* and *F*). Together, the variant structures of E533K and E554K reveal a structural basis for the disruption of E2F^TD^ binding. However, from these structures alone, it is not clear how the mutations induce fold destabilization. In the structures of Rb^P^-E2F2^TD^ Rb^P^-E2F1^TD^, both E554 and E533 form A/B subdomain-spanning salt bridges to K653 (Fig. 5*B*) (37, 38). It is possible that the loss of the salt bridge interactions contributes to the large destabilization effects observed for these variants (Fig. 3, *A*–*C*).

Missense variants within the hydrophobic core of subdomain B, such as I703F, I703V, M695I, and M704V, show reduced stability and temperature-sensitive binding to E2F1^TD^ and E7^LxCxE^ (Fig. 4, *A* and *B*). To understand how these mutations induce such effects, we crystallized M704V. The crystal structure of M704V reveals changes to the packing of the hydrophobic core of subdomain B (Fig. 5*G*). Specifically, M704V introduces new steric bulk in the form of valine’s second gamma carbon. This forces alternative rotamers of F684 and L688 through changes to a van der Waals contact network. These specific changes to sidechain orientations of F684 and L688 are not observed in any other structure of Rb^P^ and constitute a unique form of hydrophobic core packing for Rb. This structure therefore provides some insight into how M704V destabilizes Rb^P^.

For a structural example of E7^LxCxE^ inhibition, S751Y was crystallized. This variant weakens both E2F1^TD^ and E7^LxCxE^ binding relative to WT but causes the greatest reduction to E7^LxCxE^ binding of all 75 missense variants tested here. The structure of S751Y reveals that the tyrosine sidechain of the mutant sits within a hydrophobic-binding pocket used for LxCxE binding (Fig. 5*H*). Specifically, the tyrosine sits in a position to compete for the binding of a conserved I or L, which constitutes a significant part of the LxCxEx(I/L)-binding epitope (19, 39). Even though the variant tyrosine creates a snug fit into the pocket used for I/L binding, this variant is not stabilizing; instead, it causes a large destabilizing effect (ΔT_m_ = −5.4 ± 0.5 °C). In the structure of WT Rb-E7^LxCxE^, the S751 sidechain helps to stabilize the N-terminus of an alpha helix through an ST motif–stabilizing H-bond to the amide at i+3 (Fig. 5*I*) (28, 43). Loss of this known helix-stabilizing motif may be responsible for the large thermostability decrease seen for S751Y.

In summary, the crystal structures reveal several unexpected insights into the structural changes that support related biochemical findings. Overall, this work provides a comprehensive and detailed understanding of structural, protein binding, and thermostability outcomes of cancer-associated missense variants in Rb. The information provided by this study may be useful for training more accurate models to predict changes to protein binding and thermostability caused by yet unstudied variants.

## Discussion

Cancer genome sequencing has revealed detailed landscapes of somatic missense mutations (12). These can provide insights to various mechanisms of inactivation of tumor suppressor proteins. Here we select many of the recurrent mutations to the pocket domain of Rb and use biochemical methods to quantify changes to binding interactions and thermostabilities, as well as structural changes caused by key variants. The most significant finding of this data set is the identification of cancer-associated mutations causing disruptions in binding to E2F^TD^; a critical interaction with strong connections to cancer cell cycles (5). Surprisingly, we found that nearly half of the missense variants tested (35/75) showed three-fold or greater E2F1^TD^-binding inhibition at physiological temperature. Variants with the strongest effects map to the E2F^TD^-binding cleft, but others throughout the A and B subdomains show effects as well. When the same variants were examined for disruptions in binding to E7^LxCxE^, 16 variants, located primarily on the B subdomain, show three-fold, or greater, binding inhibition at physiological temperature. There is considerable overlap between the variants that affect E7^LxCxE^ binding with those that also affect E2F1^TD^ binding, and these also tend to be the variants which reduce the thermostability of Rb^P^. There are also mutations that do not have overlapping effects. Specifically, out of the 75 variants tested, only four selectively inhibited E7^LxCxE^ and not E2F1^TD^ and these effects were all under five-fold: A525G; R741C; R741S; and R763T. On the other hand, several variants strongly inhibit E2F1^TD^, by greater than 10-fold, but do not affect E7^LxCxE^: S534R; S474I; S474R; K530I; H555Y; and R656Q. Some of these variants have been observed in clinical studies. The variant H555Y was identified in a multiplexed immunofluorescent approach examining subclonal lung adenocarcinoma tumor populations from five-cell profiles; however, the authors could not justify the significance of finding this mutant (44). Two incidents of the variant R656Q were identified from a genetic screen of 150 Rb patients including one unilateral case and one bilateral case from unrelated patients (45).

Protein X-ray crystallography revealed structural bases for destabilization and binding defects for key variants: E533K; E554K; S751Y and M704V. Although, it was not clear from the structure of M704V why E2F1^TD^ and E7^LxCxE^ binding are affected by this variant, since the mutation site is distant from binding sites. A similar crystallographic study on p53 revealed that a mutation known to have temperature-sensitive DNA-binding effects causes only subtle changes to sidechain packing within the hydrophobic core of the p53 core domain (46). While Rb’s M704V mutation alters the hydrophobic core energetic ground state by reconfiguring several buried residues, these changes must also have structural consequences that extend beyond the hydrophobic core. Additional experiments are needed to elucidate the structural mechanisms through which changes to core packing translate to E2F^TD^- and E7^LxCxE^-binding inhibition by some Rb^P^ variants.

Many of the variants that we attempted to study did not express in *E. coli*. While this may seem to limit the scope of this study, we consider the variants that were measured in this study significant because they exist in the context of a folded pocket domain. We were not able to use our methods to study the most commonly observed, pathological Rb^P^ missense mutation, R661W. However, R661W has successfully expressed in other host systems where intact binding-dependent functions provide evidence of its folding (23, 26). Of the temperature-sensitive variants that we were able to study, I703F displayed the strongest effect, reducing binding six-fold more at physiological temperature (37 °C) compared with room temperature (25 °C) for E2F1^TD^. For E7^LxCxE^, 17-fold weaker binding was measured at physiological temperature relative to room temperature. Significantly, this was also the most destabilizing variant studied, as it reduced the T_m_ of the protein −6.6 °C relative to WT. Under the binding condition at physiological temperature, the temperature sensitivity for the few variants tested were reversible; however, it is very likely that under extended incubations at physiological temperature, irreversible unfolding of the pocket domain may be a predominant mechanism for loss of binding activity for many variants. Stability studies on WT Rb pocket have shown how it is susceptible to irreversible unfolding *in vitro*, such that 50% of the protein is unfolded in 30 min at physiological temperature (36). On the other hand, E2F1^TD^ and E7^LxCxE^ binding to Rb^P^ both produce stabilizing effects for Rb (36, 41). Taking this into consideration with our findings, it is likely that some missense mutations in the cancer cell have dual destabilizing effects on Rb^P^: they destabilize Rb^P^ directly yet also further destabilize Rb^P^ through disruptions to endogenous interactions at the E2F^TD^-binding site and the LxCxE-binding site.

## Experimental procedures

### Cloning, protein expression, and purification

Missense variants were cloned into a human Rb^P^ gene construct (corresponding to amino acids 380–788) (UniProt P06400) in a pGEX-4T expression vector (GenBank M21676, M97937). Primers were designed using an optimized QuickChange protocol for site-directed mutagenesis (Table S3) and ordered through a commercial vendor (IDT) (47). Plasmids were sequenced to confirm the presence of the mutations (Plasmidsaurus, Genewiz). Plasmids were transformed into BL21 DE3-pRIL–modified *E. coli* cells, grown in Terrific broth, and induced overnight with 1 mM IPTG at 25 °C. Cells were lysed using sonication and cell debris was pelleted through centrifugation. The overexpressed GST fusions in the supernatant were purified by glutathione affinity chromatography in a buffer containing 200 mM NaCl, 1 mM DTT, and 25 mM Tris–HCl (pH 8.0) and eluted using 10 mM glutathione. The fusion proteins were cleaved from the GST affinity tag using 1% TEV protease at 4 °C overnight. Proteins were then further purified by cation exchange chromatography on a heparin column in tandem with a GST trap, in order to collect residual uncut GST-Rb or cut GST tag. Protein purity was characterized by SDS-PAGE and UV 260/280.

### Fluorescence polarization

FP experiments were set up using Matrix Equalizer electronic multichannel pipettes in 20 μl reaction volumes in black untreated 384-well plates (Corning). All solutions were prepared using a buffer containing 25 mM Tris (pH 8.0), 200 mM NaCl, 1 mM DTT, and 0.1% Tween-20. Synthetic, fluorescently-labeled peptides were ordered from GenScript: TMR-E7^LxCxE^ (DLYCYEQLN), TMR-E2F1^TD^ (GEGIRDLFDCDFGDLTPLD). For the binding experiments, concentrations of 1.5 nM TMR- E2F1^TD^ and 7.5 nM TMR- E7^LxCxE^ were used with unlabeled Rb^P^. For the 25 °C experiment, samples were allowed to incubate for 2 minutes at 25 °C to ensure that binding equilibrium had been reached. FP was measured on a PHERAstar Microplate Reader using Labtech software (BMG). Reported y-axis values of millipolarization (mP) were determined from the software and calculated from the relationship: mP = 1000 × (S−G × P)/(S+G × P), such that S is the fluorescence intensity parallel to the excitation plane, P is the perpendicular fluorescence intensity, and G is the correction factor. For the 37 °C experiment, following measurement at 25 °C, the temperature of the PHERAstar instrument (with sample plate) was ramped up over a duration of 7 min to 37 °C, followed by a 2-min incubation at 37 °C before FP data was collected. For the 37 °C back to 25 °C experiment, sample plates were incubated at 25 °C, ramped up to 37 °C in an Heratherm plate incubator (Thermo Fisher Scientific) over 7 min with careful temperature monitoring by thermometer, incubated for 2 min at 37 °C, cooled on ice for 1 min, then incubated at 25 °C for 3 minutes prior to taking FP measurements. All experiments were conducted in quadruplicate. A quadratic equation was used to fit the binding data and calculate the K_d_ or apparent K_d_ in Prism 9 (GraphPad) (35). Reported errors in the K_d_ or apparent K_d_ from FP measurements are curve-fitting errors (confidence interval 95%) and error bars are SDs of four replicates. Data was collected and processed using BMG Labtech and MARS software (BMG).

### Differential scanning fluorimetry

DSF assays were performed using SYPRO orange dye (Thermo Fisher Scientific) in a 1:625 dilution in a buffer containing 200 mM NaCl, 25 mM Tris (pH 8.0), 2 mM DTT. Reactions were run in 20 μl reaction volumes with final Rb^P^ concentrations of 3 μM, 6 μM, and 9 μM. Replicates of three experiments were conducted for each condition. Experiments with different dye to protein ratios were averaged together in order to average out concentration-dependent effects (40). All experiments were performed on a QuantStudio three Real-Time PCR (Thermo Fisher Scientific) and the raw fluorescence data was fit using a Boltzmann function to calculate the melting temperature (T_m_) on Protein ThermalShift software (Thermo Fisher Scientific). Reported T_m_ values are the average values for the T_m_ across all replicates across conditions and the reported error is the calculated SD. Change in melting temperature values (ΔT_m_) were calculated by subtracting variant T_m_ from WT T_m_ values.

### Protein crystallization, data collection, and processing

Missense variants of Rb^P^ characterized by protein X-ray crystallography were first cloned into a Rb^P^ construct (Rb^380–787D616–642/S608E/S612A/S780A^) previously used to crystallize the Rb^PL^–Rb^P^ interaction (42). The E533K, E554K, M704V, and S751Y missense mutations were introduced to this specific construct because it crystalizes reproducibly. Site-directed mutagenesis was accomplished using a modified QuickChange protocol (47). Proteins were expressed from *E. coli* and purified as described previously; however, for this application, they were also further purified by size-exclusion chromatography on a Sephadex 200 column and with a buffer containing 200 mM NaCl, 25 mM Tris–HCl (pH 8.0), and 2 mM DTT. Eluate fractions were concentrated to approximately 7 mg/ml, and crystals were grown using hanging drop diffusion at 4 °C for 1 week in a 1:1 ratio with a condition containing 18% Peg 8K, 0.1 M sodium citrate, 0.1 M succinate pH 5.5, and 1M lithium chloride. Crystals were cryogenically stabilized in a solution containing 20% glycerol and flash frozen in liquid nitrogen. X-ray diffraction data was collected at ALS BL5.0.1. Data were indexed and integrated with XDS (48) in the space group R3:H and merged and scaled with AIMLESS (49). Molecular replacement solutions were found using PHASER (50), with the search model PDB ID: 4ELL (42). Atomic models were built and modified using COOT (51) and refined using default parameters in PHENIX (52). Structure coordinates and maps were deposited to the Protein Data Bank with the following codes: Rb E533K (9DHU); Rb E554K (9DHF); Rb M704V (9DGK); Rb S751Y (9DHC).

## Data availability

All data are available in the main text or the supplementary materials. Protein X ray structures have been deposited and are available from the Protein Data Bank under the following accession numbers: Rb E533K (PDB: 9DHU) Rb E554K (PDB: 9DHF) Rb M704V (PDB: 9DGK) Rb S751Y (PDB: 9DHC).

## Supporting information

This article contains supporting information.

## Conflict of interest

The authors declare that they have no conflicts of interest with the contents of this article.
