# Supplementary material for: Structural and functional analysis of cancer-associated missense variants in the retinoblastoma protein pocket domain
Source: J Biol Chem. 2025 Feb 10;301(3):108284. doi: 10.1016/j.jbc.2025.108284 (PMC11931385; doi:10.1016/j.jbc.2025.108284)
Supplement: Supporting information [file mmc1.docx]

**Supporting Information**

Structural and functional analysis of cancer-associated missense variants in the retinoblastoma protein pocket domain

Anthony Castro^1†^, Alfredo Ruiz-Rivera^1†^, Chad C. Moorman^1†‡^, Emma R. Wolf-Saxon^1†^, Hailey N. Mims^1^, Vanessa I. Vasquez Meza^1^, Matthew A. Rangel^1^, Marcos M. Loera^1§^, Ian C. Bond^1^, Seth B. Buchanan^1^, Estela Villarreal^1^, Sarvind Tripathi^2^, Seth M. Rubin^2^, and Jason R. Burke^1*^

^1^Department of Chemistry and Biochemistry, California State University San Bernardino, San Bernardino, California 92407, USA.

^2^Department of Chemistry and Biochemistry, University of California, Santa Cruz, CA 95064, USA

^†^These authors contributed equally to this work

^‡^Current address: Department of Chemistry and Biochemistry, University of California, Irvine, CA 92697, USA

^§^Current address: Osteopathic Medical School, Kansas City University, Kansas City, MO 94106, USA

*Corresponding Author:

Jason R. Burke

Email: [jason.burke@csusb.edu](mailto:jason.burke@csusb.edu)

Phone: +1-909-537-5338

Table of Contents

| Figure S1 | Missense variants of human Rb used or attempted in this study | pg. S2 |
| --- | --- | --- |
| Figure S2 | FP data for variants E440K, S443L, Q444L, Q444R, R451C, R451H, R451S, K462E, K462T, S463L, S474I, S474N, S474R, A488A, A490S | pg. S3 |
| Figure S3 | FP data for variants A490T, A490V, E492Q, Q504E, A525G, K530I, E533K, S534R, E539D, M546I, K548N, L550I, E554K, H555Y, R556Q | pg. S4 |
| Figure S4 | FP data for variants L572I, D584Y, H585Y, S588F, P595L, D604G, Y606C, R611S, S612P, T620M, R621C, R621H, R621P, R621S, T638S | pg. S5 |
| Figure S5 | FP data for variants S648T, V654L, R656Q, R656W, T664A, R668C, R668H, S671F, H673P, H673Y, E675Q, I 679F, H686Y, T687I, E693A | pg. S6 |
| Figure S6 | FP data for variants M695I, D697E, H699P, I703F, I703V, M704V, T738I, R741C, R741H, R741S, S751C, S751Y, V754G, M761L, R763T | pg. S7 |
| Figure S7 | Venn diagram comparison of mutations that increase K_d (app)_ values for Rb^P^-E2F1^TD^ at 25°C vs. 37°C | pg. S8 |
| Table S1 | Comparison of K_d (app)_ values for Rb^P^-E2F1 measured at different temperatures for wild type, E554K, I703F, M704V, S751Y | pg. S9 |
| Table S2 | Comparison of K_d (app)_ values for Rb^P^-E7^LxCxE^ measured at different temperatures for wild type, E554K, I703F, M704V, S751Y | pg. S10 |
| Table S3 | DNA primers used in this study for site-directed mutagenesis | pg. S11-S13 |

**Figure S1. Missense variants of human Rb used or attempted in this study.** Each missense variant was independently cloned, expressed and evaluated as a GST-Rb^P^ fusion protein from *e coli*. Missense variants that failed to produce soluble protein from this system are shown as grey bars (n=37). The variants that did express in the host system and underwent further biochemical characterizations are shown as black bars (n=75). For each variant, substitutions (numbers of incidents in patients) are taken from the COSMIC somatic cancer mutation dataset and are shown on the y-axis. Variants that have neither grey nor black bars are taken from the cBioPortal data set; these were successfully expressed and characterized in this study.

Fig. S2. FP data for variants E440K, S443L, Q444L, Q444R, R451C, R451H, R451S, K462E, K462T, S463L, S474I, S474N, S474R, A488A and A490S. Error bars are standard deviations of 4 replicates.

Fig. S3. FP data for variants A490T, A490V, E492Q, Q504E, A525G, K530I, E533K, S534R, E539D, M546I, K548N, L550I, E554K, H555Y and R556Q. Error bars are standard deviations of 4 replicates.

**Fig. S4.** FP data for variants L572I, D584Y, H585Y, S588F, P595L, D604G, Y606C, R611S, S612P, T620M, R621C, R621H, R621P, R621S and T638S. Error bars are standard deviations of 4 replicates.

**Fig. S5.** FP data for variants S648T, V654L, R656Q, R656W, T664A, R668C, R668H, S671F, H673P, H673Y, E675Q, I 679F, H686Y, T687I and E693A. Error bars are standard deviations of 4 replicates.

**Fig S6**. FP data for variants. FP data for variants M695I, D697E, H699P, I703F, I703V, M704V, T738I, R741C, R741H, R741S, S751C, S751Y, V754G, M761L and R763T. Error bars are standard deviations of 4 replicates.

Figure S7. Venn diagram comparison of mutations that increase K_d (app)_ values by 3-fold or greater for Rb^P^-E2F1^TD^ at 25°C vs. 37°C. Mutations that increase K_d (app)_ values by 3-fold or greater at both temperatures are shown in the overlap.

|  | Rb^P^ - E2F1  K_d (app)_ 25 °C  (1^st^ measurement) | Rb^P^ - E2F1  K_d (app)_ 37 °C  (2^nd^ measurement) | Rb^P^ - E2F1  K_d (app)_ 25 °C  (3^rd^ measurement) |
| --- | --- | --- | --- |
| Wild type | 14 ± 3* nM | 22 ± 4 nM | 17 ± 5 nM |
| E554K | 1167 ± 98 nM | 3266 ± 722 nM | 1737 ± 224 nM |
| I703F | 63 ± 12 nM | 396 ± 12 nM | 94 ± 13 nM |
| M704V | 41 ± 8 nM | 129 ± 37 nM | 46 ± 9 nM |
| S751Y | 33 ± 5 nM | 127 ± 25 nM | 28 ± 8 nM |

Table S1. Comparison of K_d (app)_ values for Rb^P^-E2F1 measured at different temperatures for wild type, E554K, I703F, M704V, S751Y (*denotes K_d_ value). Data from columns 2 (1^st^ measurement) and 3 (2^nd^ measurement) are reprinted from Table 1 for comparative purposes.

|  | Rb^P^- E7^LxCxE^  K_d (app)_ 25 °C  (1^st^ measurement) | Rb^P^- E7^LxCxE^  K_d (app)_ 37 °C  (2^nd^ measurement) | Rb^P^- E7^LxCxE^  K_d (app)_ 25 °C  (3^rd^ measurement) |
| --- | --- | --- | --- |
| Wild type | 5 ± 1* nM | 12 ± 1 nM | 7 ± 1 nM |
| E554K | 12 ± 3 nM | 48 ± 9 nM | 24 ± 2 nM |
| I703F | 16 ± 2 nM | 275 ± 26 nM | 53 ± 6 nM |
| M704V | 18 ± 4 nM | 51 ± 16 nM | 14 ± 1 nM |
| S751Y | 127 ± 8 nM | 420 ± 38 nM | 119 ± 12 nM |

Table S2. Comparison of K_d (app)_ values for Rb^P^-E7^LxCxE^ measured at different temperatures for wild type, E554K, I703F, M704V, S751Y (*denotes K_d_ value). Data from columns 2 (1^st^ measurement) and 3 (2^nd^ measurement) are reprinted from Table 1 for comparative purposes.

Table S3. DNA primers used in this study for site-directed mutagenesis

| MUTATION | FORWARD PRIMER | REVERSE PRIMER |
| --- | --- | --- |
| E440K | gttgtgtcaaaattggatcacagcgatacaaac | gctgtgatccaattttgacacaaccctgtcc |
| S443L | gaaattggattacagcgatacaaacttgg | cgctgtaatccaatttcgacacaaccctg |
| Q444L | ggatcactgcgatacaaacttggag | gtatcgcagtgatccaatttcgac |
| Q444R | ggatcacggcgatacaaacttggag | gtatcgccgtgatccaatttcgac |
| G449E | gatacaaacttgaagttcgcttgtattaccg | caagcgaacttcaagtttgtatcgctgtgatcc |
| R451C | cttggagtttgcttgtattaccgagtaatgg | cggtaatacaagcaaactccaagtttgtatcgc |
| R451H | cttggagttcacttgtattaccgagtaatgg | cggtaatacaagtgaactccaagtttgtatcgc |
| R451S | cttggagttagcttgtattaccgagtaatgg | cggtaatacaagctaactccaagtttgtatcgc |
| Y454C | cgcttgtattgccgagtaatggaatccatgc | ccattactcggcaatacaagcgaactcc |
| Y454H | cgcttgtatcaccgagtaatggaatccatgc | ccattactcggtgatacaagcgaactcc |
| M457R | cgagtaagggaatccatgcttaaatcag | catggattcccttactcggtaatacaagc |
| L461P | ggaatccatgcctaaatcagaagaagaacg | cttctgatttaggcatggattccattactcgg |
| K462E | ggaatccatgcttgaatcagaagaagaacg | cttctgattcaagcatggattccattactcgg |
| K462T | ggaatccatgcttacatcagaagaagaacg | cttctgatgtaagcatggattccattactcgg |
| S463L | ccatgcttaaattagaagaagaacgattatcc | cttcttctaatttaagcatggattccattac |
| S474I | cattcaaaattttatcaaacttctgaatgacaacatttttc | cagaagtttgataaaattttgaatggataatcgttc |
| S474N | ccattcaaaattttaacaaacttctgaatgacaacatttttc | cagaagtttgttaaaattttgaatggataatcgttc |
| S474R | ccattcaaaattttagaaaacttctgaatgacaacatttttc | cagaagttttctaaaattttgaatggataatcgttc |
| H483R | caacatttttcgtatgtctttattggcgtgcgctcttg | ccaataaagacatacgaaaaatgttgtcattcagaag |
| H483Y | caacattttttatatgtctttattggcgtgcgctcttg | ccaataaagacatataaaaaatgttgtcattcagaag |
| A488E | ctttattggagtgcgctcttgaggttgtaatggcc | caagagcgcactccaataaagacatatg |
| A488V | ctttattggtgtgcgctcttgaggttgtaatggcc | caagagcacacaccaataaagacatatg |
| A490S | ggcgtgctctcttgaggttgtaatggccac | caacctcaagagagcacgccaataaagacatatg |
| A490T | ggcgtgcactcttgaggttgtaatggccac | caacctcaagagtgcacgccaataaagacatatg |
| A490V | ggcgtgcgttcttgaggttgtaatggccac | caacctcaagaacgcacgccaataaagacatatg |
| L491P | cgtgcgctcctgaggttgtaatggccacatatagcag | cattacaacctcaggagcgcacgccaataaagac |
| E492K | cgtgcgctcttaaggttgtaatggccacatatagc | ccattacaaccttaagagcgcacgccaataaagac |
| E492Q | cgtgcgctcttcaggttgtaatggccacatatagc | ccattacaacctgaagagcgcacgccaataaagac |
| Q504E | gtacatctgagaatcttgattctggaacagatttg | gaatcaagattctcagatgtacttctgctatatgtg |
| P515S | gatttgtctttctcatggattctgaatgtgc | gaatccatgagaaagacaaatctgttccagaatc |
| L521P | ctgaatgtgcctaatttaaaagcctttgatttttac | cttttaaattaggcacattcagaatccatggg |
| A525G | cttaatttaaaaggctttgatttttacaaagtgatcg | gtaaaaatcaaagccttttaaattaagcacattcag |
| K530I | gatttttacatagtgatcgaaagttttatcaaagc | cgatcactatgtaaaaatcaaaggcttttaaattaag |
| I532N | caaagtgaacgaaagttttatcaaagcagaag | ctttcgttcactttgtaaaaatcaaag |
| E533K | gtgatcaaaagttttatcaaagcagaaggcaac | gataaaacttttgatcactttgtaaaaatcaaag |
| S534R | gtgatcgaaagatttatcaaagcagaaggcaac | gataaatctttcgatcactttgtaaaaatcaaag |
| E539D | caaagcagacggcaacttgacaagagaaatg | gttgccgtctgctttgataaaactttcg |
| M546I | caagagaaattataaaacatttagaacgatgtgaacatcg | ctaaatgttttataatttctcttgtcaagttgccttctg |
| K548N | gagaaatgataaaccatttagaacgatgtgaacatcg | gttctaaatggtttatcatttctcttgtcaagttgc |
| L550I | gataaaacatatagaacgatgtgaacatcgaatcatgg | catcgttctatatgttttatcatttctcttgtcaag |
| C553Y | gaacgatatgaacatcgaatcatggaatcccttg | gattcgatgttcatatcgttctaaatgttttatc |
| E554K | gaacgatgtaaacatcgaatcatggaatcccttg | gattcgatgtttacatcgttctaaatgttttatc |
| H555Y | gatgtgaatatcgaatcatggaatcccttgcatg | catgattcgatattcacatcgttctaaatg |
| R556Q | gtgaacatcaaatcatggaatcccttgcatg | catgatttgatgttcacatcgttctaaatg |
| A562E | gaatcccttgaatggctctcagattcacctttatttg | ctgagagccattcaagggattccatgattcgatg |
| A562P | gaatcccttccatggctctcagattcacctttatttg | ctgagagccatggaagggattccatgattcgatg |
| S567L | ctctcagatttacctttatttgatcttattaaac | caaataaaggtaaatctgagagccatgcaaggg |
| L572F | ctttatttgattttattaaacaatcaaaggacc | gtttaataaaatcaaataaaggtgaatctg |
| L572I | ctttatttgatattattaaacaatcaaaggacc | gtttaataatatcaaataaaggtgaatctg |
| D584Y | ggaccaacttatcaccttgaatctgcttgtc | caaggtgataagttggtccttctcggtc |
| H585Y | ccaactgattaccttgaatctgcttgtcctc | gattcaaggtaatcagttggtccttctcggtc |
| S588F | caccttgaatttgcttgtcctcttaatcttcc | ggacaagcaaattcaaggtgatcagttggtcc |
| P595L | cttaatcttcttctccagaataatcacactgcag | gattattctggagaagaagattaagaggacaagcag |
| D604G | ctgcagcaggtatgtatctttctcctgtaagatc | gaaagatacatacctgctgcagtgtgattattc |
| Y606C | cagatatgtgtctttctcctgtaagatctccaaag | ggagaaagacacatatctgctgcagtgtgattattc |
| R611S | ctcctgtaagttctccaaagaaaaaaggttc | ctttggagaacttacaggagaaagatacatatc |
| S612P | ctgtaagacctccaaagaaaaaaggttcaac | ctttggaggtcttacaggagaaagatac |
| T620M | gttcaactatgcgtgtaaattctactgcaaatgc | gaatttacacgcatagttgaaccttttttctttg |
| R621C | caactacgtgtgtaaattctactgcaaatgcagag | gaatttacacacgtagttgaaccttttttctttg |
| R621H | caactacgcatgtaaattctactgcaaatgcagag | gaatttacatgcgtagttgaaccttttttctttg |
| R621P | caactacgcctgtaaattctactgcaaatgcagag | gaatttacaggcgtagttgaaccttttttctttg |
| R621S | caactacgagtgtaaattctactgcaaatgcagag | gaatttacactcgtagttgaaccttttttctttg |
| T638S | ccttccagtcccagaagccattgaaatctacc | ggcttctgggactggaaggctgaggttgcttg |
| L647R | cctctcgttcactgttttataaaaaagtgtatcggc | cagtgaacgagaggtagatttcaatg |
| V654L | gttttataaaaaactgtatcggctagcctatctc | ctagccgatacagttttttataaaacagtgaaagagag |
| R656Q | gtgtatcagctagcctatctccggctaaatac | gataggctagctgatacacttttttataaaacag |
| R656W | gtgtattggctagcctatctccggctaaatac | gataggctagccaatacacttttttataaaacag |
| R661Q | cctatctccagctaaatacactttgtgaacgc | gtatttagctggagataggctagccgatacac |
| R661W | cctatctctggctaaatacactttgtgaacgc | gtatttagccagagataggctagccgatacac |
| T664A | ggctaaatgcactttgtgaacgccttctgtc | cacaaagtgcatttagccggagataggctag |
| R668C | ctttgtgaatgccttctgtctgagcacccagaattag | cagaaggcattcacaaagtgtatttagccggag |
| R668H | ctttgtgaacaccttctgtctgagcacccagaattag | gacagaaggtgttcacaaagtgtatttagccggag |
| S671F | ccttctgtttgagcacccagaattagaac | ggtgctcaaacagaaggcgttcacaaagtg |
| H673P | gtctgagcccccagaattagaacatatcatctgg | ctaattctgggggctcagacagaaggcgttcacaaag |
| H673Y | ctgtctgagtacccagaattagaacatatcatctgg | ctaattctgggtactcagacagaaggcgttcacaaag |
| E675Q | gcacccacaattagaacatatcatctggaccc | gatatgttctaattgtgggtgctcagacagaaggc |
| I679F | gaattagaacatttcatctggacccttttccagc | ccagatgaaatgttctaattctgggtgctc |
| I680T | gaacatatcacctggacccttttccagcacac | gggtccaggtgatatgttctaattctgggtg |
| H686Y | cttttccagtacaccctgcagaatgagtatgaactc | gcagggtgtactggaaaagggtccagatgatatg |
| T687I | ccagcacatcctgcagaatgagtatgaactc | ctgcaggatgtgctggaaaagggtccagatg |
| L688P | gcacaccccgcagaatgagtatgaactcatg | ctcattctgcggggtgtgctggaaaagggtccag |
| E693A | gagtatgcactcatgagagacaggcatttggacc | gagtatgcactcatgagagacaggcatttggacc |
| L694P | gagtatgaacccatgagagacaggcatttggacc | gagtatgaacccatgagagacaggcatttggacc |
| M695I | gaactcattagagacaggcatttggacc | cctgtctctaatgagttcatactcattctgc |
| M695K | gaactcaagagagacaggcatttggacc | cctgtctctcttgagttcatactcattctgc |
| D697E | catgagagagaggcatttggaccaaattatg | caaatgcctctctctcatgagttcatactcattc |
| R698S | gagagacagccatttggaccaaattatgatgtg | gtccaaatggctgtctctcatgagttcatactc |
| R698W | gagagactggcatttggaccaaattatgatgtg | gtccaaatgccagtctctcatgagttcatactc |
| H699P | gagacaggcctttggaccaaattatgatgtg | ggtccaaaggcctgtctctcatgagttcatac |
| D701N | ggcatttgaaccaaattatgatgtgttccatg | cataatttggttcaaatgcctgtctctcatgag |
| D701V | ggcatttggtccaaattatgatgtgttccatg | cataatttggaccaaatgcctgtctctcatgag |
| D701Y | ggcatttgtaccaaattatgatgtgttccatg | cataatttggtacaaatgcctgtctctcatgag |
| Q702K | catttggacaaaattatgatgtgttccatgtatgg | catcataattttgtccaaatgcctgtctctcatgag |
| I703F | ggaccaatttatgatgtgttccatgtatggcatatg | cacatcataaattggtccaaatgcctgtctctc |
| I703V | ggaccaagttatgatgtgttccatgtatggcatatg | cacatcataacttggtccaaatgcctgtctctc |
| M704V | gaccaaattgtgatgtgttccatgtatggcatatg | gaacacatcacaatttggtccaaatgcctgtctc |
| C706F | caaattatgatgttttccatgtatggcatatgcaaagtg | catacatggaaaacatcataatttggtccaaatgcctgtc |
| C706Y | caaattatgatgtattccatgtatggcatatgcaaagtg | catacatggaatacatcataatttggtccaaatgcctgtc |
| M708K | gtgttccaagtatggcatatgcaaagtgaagaatatag | catatgccatacttggaacacatcataatttggtc |
| I724N | caaaatcaatgtaacagcatacaaggatc | gctgttacattgattttgaatttaaggtctatattc |
| I724S | caaaatcagtgtaacagcatacaaggatc | gctgttacactgattttgaatttaaggtctatattc |
| T738I | gttcaggagatattcaaacgtgttttgatcaaag | cacgtttgaatatctcctgaacagcatgagg |
| R741C | cattcaaatgtgttttgatcaaagaagaggag | gatcaaaacacatttgaatgtctcctgaacagcatg |
| R741H | cattcaaacatgttttgatcaaagaagaggag | gatcaaaacatgtttgaatgtctcctgaacagcatg |
| R741S | cattcaaaagtgttttgatcaaagaagaggag | gatcaaaacacttttgaatgtctcctgaacagcatg |
| S751C | gtatgattgtattatagtattctataactcggtc | gaatactataatacaatcatactcctcttctttgatc |
| S751Y | gtatgattatattatagtattctataactcggtc | gaatactataatataatcatactcctcttctttgatc |
| V754G | ctattataggattctataactcggtcttcatgc | gttatagaatcctataatagaatcatactcctcttc |
| Y756C | gtattctgtaactcggtcttcatgcagag | ccgagttacagaatactataatagaatcatac |
| S758L | gtattctataacttggtcttcatgcagagactg | catgaagaccaagttatagaatactataatagaatc |
| M761L | gtcttcctgcagagactgaaaacaaatattttg | gtctctgcaggaagaccgagttatagaatac |
| R763T | catgcagacactgaaaacaaatattttgcag | gttttcagtgtctgcatgaagaccgagttatag |
